# Clinical Practice Variation Among Pediatric Rheumatologists Treating Kawasaki Disease: Results of a North American Survey

**DOI:** 10.3390/children12121695

**Published:** 2025-12-16

**Authors:** Daniel Ibanez, Bianca Lang, Julia Shalen, Ali Yalcindag, Linda Wagner-Weiner, Kenneth N. Schikler, Shoghik Akoghlanian, Hulya Bukulmez, Kristen Hayward, Laura Berbert, Sivia Lapidus, Andrea A. Ramirez, Cagri Yildirim-Toruner

**Affiliations:** 1 Division of Immunology, Boston Children’s Hospital, Harvard Medical School, Boston, MA 02115, USA; dji35@miami.edu (D.I.);; 2 Division of Rheumatology, IWK Health, Dalhousie University, Halifax, NS B3K 6R8, Canada; balang@dal.ca; 3 Division of Pediatric Allergy, Immunology and Rheumatology, School of Medicine, Johns Hopkins University, Baltimore, MD 21287, USA; 4 Hasbro Children’s Hospital, The Warren Alpert Medical School of Brown University, Providence, RI 02903, USA; 5 Comer Children’s Hospital, The University of Chicago, Chicago, IL 60637, USA; 6 Norton Children’s Hospital, University of Louisville School of Medicine, Louisville, KY 40202, USA; 7 Nationwide Children’s Hospital, The Ohio State University College of Medicine, Columbus, OH 43205, USA; 8 Division of Pediatric Rheumatology, The MetroHealth System, Case Western Reserve University, Cleveland, OH 44109, USA; 9 Seattle Children’s Hospital, University of Washington School of Medicine, Seattle, WA 98105, USA; 10 Division of Pediatric Rheumatology, The Joseph M. Sanzari Children’s Hospital, Hackensack Meridian School of Medicine, Hackensack, NJ 07601, USA; 11 Division of Rheumatology, Texas Children’s Hospital, Baylor College of Medicine, Houston, TX 77030, USA

**Keywords:** Kawasaki disease, IVIG-refractory, coronary artery aneurysm, treatment variation, consensus treatment plan, primary intensification

## Abstract

**Background**: The best treatment for children with KD who fail to respond to the first dose of IVIG (refractory KD) is currently unknown. The purpose of this study was to determine treatment practices of pediatric rheumatologists in North America who manage IVIG-refractory KD. **Methods**: A 34-item web-based survey was sent to 102 randomly selected members of the Childhood Arthritis and Rheumatology Research Alliance (CARRA). The anonymous survey addressed the use of primary intensification as well as the treatment of IVIG-refractory KD. **Results**: The response rate was 82%; 56% (all pediatric rheumatologists) completed the survey. Primary intensification was used for macrophage activation syndrome (MAS), KD shock, and those at high risk for coronary artery aneurysms (CAAs) by 84%, 76% and 52% of responders, respectively, with corticosteroids (CSs) used most frequently. For IVIG-refractory KD without CAA, a second dose of IVIG was used most often (63% alone; 23% plus CS). With non-giant CAAs, only 15% used a second IVIG alone, 40% used IVIG plus CS, and 35% took infliximab, usually with CS/IVIG. With giant CAA, treatments used most frequently were CS, a second IVIG, and infliximab (91%, 69%, and 58%, respectively), usually as combinations of two or more medications. **Conclusions**: Treatment of IVIG-refractory KD varies significantly among North American pediatric rheumatologists, particularly in the presence of CAAs. Our findings emphasize the need for research to identify the most effective therapy for this KD subgroup. The current use of primary intensification and the presence and size of the CAA will need to be considered as consensus treatment plans are developed.

## 1. Introduction

Kawasaki Disease (KD), a systemic vasculitis, is a leading cause of acquired heart disease in children, primarily due to the morbidity and mortality associated with the development of coronary artery aneurysms (CAAs) [1]. Prompt treatment with IVIG has significantly decreased the incidence of CAAs in KD. However, 10–20% of patients with KD fail to respond to treatment with a first dose of IVIG and are termed IVIG-refractory KD [2,3]. Recognizing this subgroup of patients is essential since they are at higher risk for CAA development and require additional treatment to control ongoing inflammation. Due to the relative rarity of this condition, there is a paucity of evidence-based studies following adequate patient numbers longitudinally to establish optimal treatment regimens for refractory KD [4].

The most frequently recommended treatment, included in guidelines worldwide, is a second dose of IVIG [3,5,6]. Infliximab has been used as an alternative treatment for patients who have not responded to their first dose of IVIG, with several studies demonstrating its safety, as well as a shorter duration of fever and hospitalization compared to a second dose of IVIG. Nevertheless, a randomized, multicenter comparative effectiveness trial (KIDCARE) that compared infliximab (10 mg/kg) to a second dose of IVIG for refractory KD found no difference in CA outcome or inflammatory markers [7]. Corticosteroids (CSs), with or without a second dose of IVIG, have also been used in patients with IVIG-refractory KD [7,8,9,10]. A retrospective study comparing a second dose of IVIG alone, a second dose of IVIG plus prednisolone, and prednisolone alone reported improved outcomes with the combination of CSs plus IVIG, including fewer CAAs and lower retreatment rates compared with either IVIG or CSs alone [11]. Anakinra, an Il-1 receptor antagonist, has recently been studied in refractory KD. Small phase I/II clinical trials have shown promising results, including a good safety profile, and a phase III multicenter comparative trial of anakinra versus IVIG retreatment is currently underway (ClinicalTrials.gov ID NCT04656184) [12].

The choice of treatment for refractory KD may be affected by the initial treatment received by a patient. Although the initial management of uncomplicated KD is fairly standard, there is increasing awareness that IVIG combined with adjunctive therapy at diagnosis, termed primary intensification, may lead to improved outcomes in patients with severe disease and those at high risk for CAAs [1,13]. CSs are recommended for primary intensification in several recent guidelines, which have been supported by the Japanese randomized controlled RAISE trial and the follow-up post-RAISE study, which both demonstrated improved CA outcomes with CS [3,5,6,9,13,14]. A role for infliximab as primary intensification is supported by retrospective studies, which have shown more frequent CAA regression and less progression in CAA size in patients with CAAs at diagnosis who received infliximab [15,16]. A randomized controlled trial (RCT) of IVIG plus infliximab (5 mg/kg) versus IVIG alone showed reduced fever duration after infliximab, but no reduction in IVIG resistance or CAAs [17]. A Japanese study also suggested a possible role of cyclosporin A for primary intensification in high-risk KD [18].

Despite efforts to determine optimal treatment, variability in care is common, and outcomes remain suboptimal. It is difficult to conduct pediatric randomized controlled trials (RCTs) due to the low incidence of pediatric rheumatic diseases. Cost-related constraints and limited resources remain barriers to providing a robust evidence base for decision-making. Comparative effectiveness research (CER) is an alternative approach for situations where RCTs are not feasible and allows a real-world perspective. The Childhood Arthritis and Rheumatology Research Alliance (CARRA), an investigator-led collaborative research organization, develops consensus treatment plans (CTPs) using consensus methodology and existing scientific evidence with the intent to reduce treatment variation and standardize reporting of outcome measures, thereby facilitating CER [19]. Since 2009, CARRA has developed thirteen CTPs focused on pediatric rheumatic diseases with the aim of reducing treatment variability and promoting the conduct of CER utilizing the CARRA Registry (CARRA CTP website: Consensus Treatment Plans|CARRA, https://carragroup.org/research/consensus-treatment-plans/, accessed on 15 June 2025).

To guide the development of IVIG-refractory KD CTPs, the CARRA KD workgroup developed a survey to assess the treatment practices of pediatric rheumatologists in North America (NA) who manage IVIG-refractory KD. Our survey also sought to determine if the COVID-19 pandemic had led to changes in KD treatment.

## 2. Materials and Methods

### 2.1. Study Design

When joining CARRA, members are invited to participate in one or more disease-specific workgroups, including the CARRA KD workgroup. In March 2022, the KD workgroup undertook an anonymous cross-sectional electronic web-based survey (www.SurveyMonkey.com) of 102 randomly selected CARRA physician members. Trainee (fellow and resident) CARRA members were excluded. The survey was distributed by email between 24 March and 20 April 2022. Although all responses were anonymous, respondents were tracked, and three reminders were sent to non-responders to encourage survey completion until an 80% response rate was reached. Early exit points from the survey were provided for respondents who had not treated a patient with IVIG-refractory KD within the prior three years or otherwise wanted to leave the survey. This study followed usual CARRA survey methods for assessing variation in treatment for rare pediatric rheumatic diseases.

### 2.2. Survey

Members of the CARRA KD workgroup developed a 34-item survey that included multiple-choice questions with open-ended options, as well as clinical vignettes. Clinical scenarios and questions for the survey were initially developed at an in-person CARRA meeting in 2019 (Louisville, KY, USA). Subsequently, a smaller core group worked on and finalized the survey over virtual Zoom meetings during the COVID-19 pandemic (Supplementary Materials). Beta testing was performed by four pediatric rheumatologists who were not involved in the survey development, and the survey was modified accordingly. The survey collected information on the respondents’ specialty training, their number of years in practice, and the number of IVIG-refractory KD patients seen. It also asked about the medical services involved in KD care at their institution, whether an institution-specific KD treatment protocol existed, and the availability and timing of echocardiography. Participants answered questions regarding their use of primary intensification for KD patients deemed to be at higher risk of developing CAAs or having severe illnesses, including circumstances that would lead to such treatment, as well as therapies used. Respondents were asked to define refractory KD and indications for further treatment after failed response to initial IVIG, including for patients with normal CAs, non-giant CAAs or giant CAAs, and whether any high-risk features of KD would have led to a different treatment choice.

Medication choices for the initial treatment of KD and treatment of refractory KD were explored using clinical vignettes, where respondents were asked to indicate their preferred treatment for the presented patients. The survey emphasized that the cases included in the clinical vignettes had an established diagnosis of KD and did not have multisystem inflammatory syndrome in children (MIS-C). Infection was excluded in the clinical vignettes with refractory KD, and fever was present 48 h after completion of IVIG, to ensure that the cases presented would meet the definition of refractory KD for all respondents. Respondents were also asked if and how their management of KD had changed after the COVID-19 pandemic.

The survey was approved by the CARRA CTP Advisory Committee. The study was approved by the Institutional Review Board at the University of Chicago (IRB approval #: IRB22-0292), and the requirement for informed consent was waived. For analysis, data were transferred to a REDCap (Research Electronic Data Capture) database hosted at Boston Children’s Hospital. Descriptive statistics were used to present the data.

## 3. Results

### 3.1. Demographic Results

**Figure 1:**
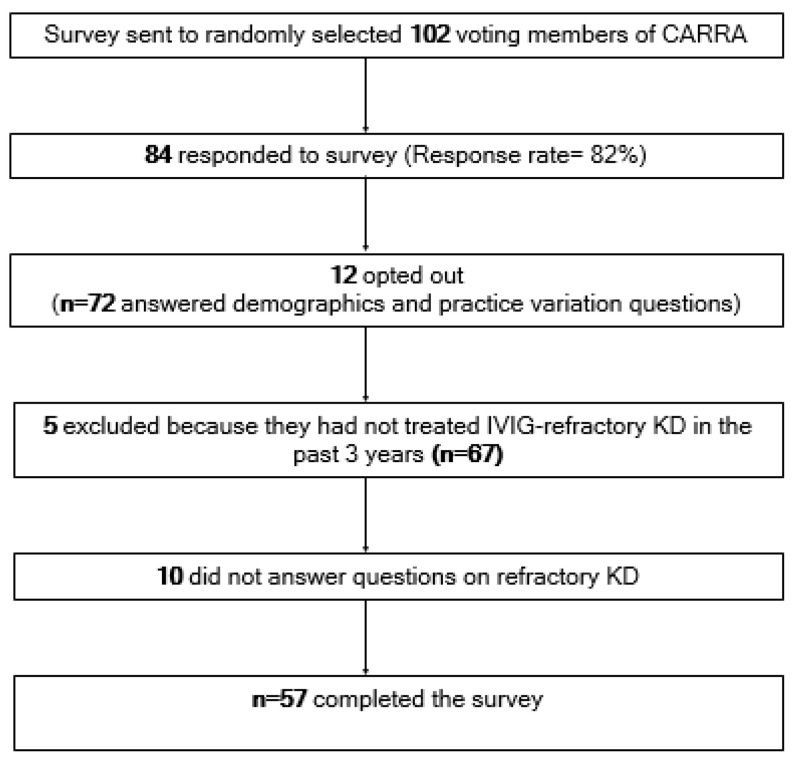
Survey response and completion.

In total, 84 of 102 CARRA physicians, all pediatric rheumatologists, responded to the survey (response rate: 82%). Twelve respondents opted out of the survey; seventy-two pediatric rheumatologists completed the survey section on demographics and practice variation. Five respondents were excluded because they had not treated a patient with IVIG-refractory KD in the past 3 years. Sixty-seven responded to questions about the initial treatment of KD. Ten did not respond to questions on refractory KD; fifty-seven completed the survey (Figure 1). Incomplete responses led to lower numbers of respondents for some questions, as it was not mandatory to answer all questions. Clinical experience varied, with 29% of respondents in practice for <5 years, 28% for 6–10 years, and 43% for more than 10 years. In the prior 3 years, 42% of respondents estimated treating 6–10 patients with IVIG-refractory KD, 36% managed 1–5, 15% treated 11–20, and 8% managed more than 20 patients.

### 3.2. Patterns of KD Care and Initial Treatment of KD Without High-Risk Features

Differences were observed in care patterns, including physicians’ roles in managing KD patients. Hospitalists or general pediatricians were the primary providers for patients with KD per most respondents (81%). Other specialty services usually provided consultant care, including rheumatology (86%), cardiology (79%), and infectious disease (61%). All respondents reported access to echocardiograms; however, 13% did not have access prior to the initial treatment. No institution-specific protocol for refractory KD was reported by 63% of respondents, while 28% had a treatment protocol and 9% were uncertain. All respondents involved in the initial management of KD patients would use IVIG (2g/kg) and ASA as initial treatments for a KD patient without CAAs or high-risk features. Moderate-dose ASA (30–50 mg/kg/d) was used most often (45%), followed by high-dose (>80 mg/kg/d) (33%) and low-dose ASA (3–5 mg/kg/d) (15%). 

### 3.3. Definition of IVIG-Refractory KD

Fifty-four percent of respondents defined refractory KD based on the presence of a temperature >100.4 F (38 degrees C) at least 36 h after completion of the first dose of IVIG. Thirty-three percent of respondents would make the diagnosis of refractory KD at 24 h after completion of the first dose of IVIG, while 11% did not make the diagnosis until 48 h after IVIG completion. In the absence of fever following the completion of IVIG, the need for additional therapy would be considered by 79% of respondents in the presence of a CAA alone and by 47% of respondents if the CRP continued to increase.

### 3.4. Primary Intensification Treatment of KD

**Figure 2:**
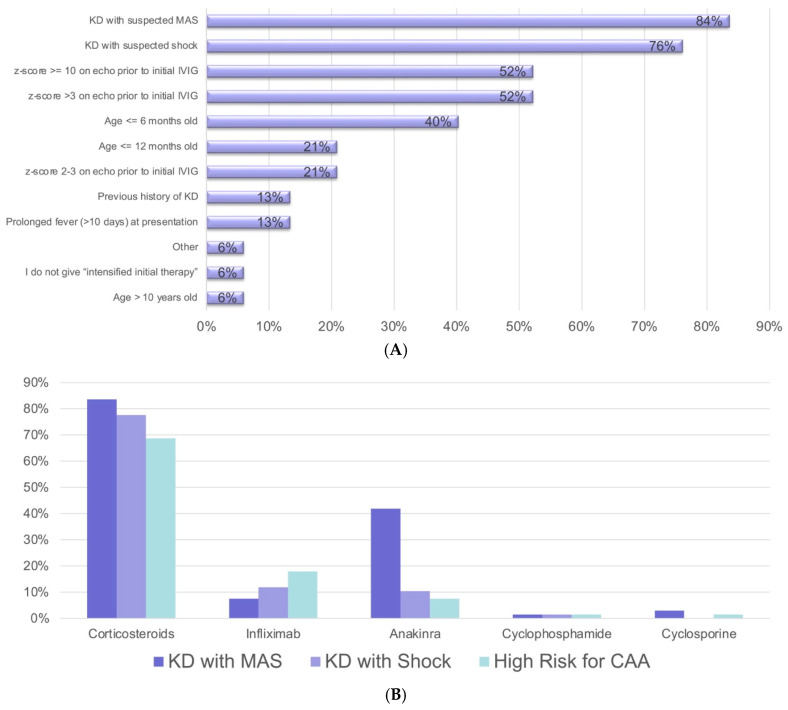
(**A**) Indications for primary intensification treatment of KD. (**B**) Medication use for primary intensification treatment of KD.

Most respondents reported using primary intensification therapy for patients with KD complicated by macrophage activation syndrome (MAS) (84%) and KD shock syndrome (KDSS) (76%). The next most common indications for primary intensification were an abnormal baseline echo with a Z-score above 3 (52%), age 6 months or younger (40%), age 12 months or younger (27%), and a Z-score of 2–3 on the baseline echo (21%). Less commonly reported indications for intensification were a prior history of KD (13%), prolonged fever (>10 days) at presentation (13%), and age more than 10 years (6%). Only 6% of respondents reported never using primary intensification treatment in KD patients. Corticosteroids (CSs) were used most frequently for intensification, regardless of the indication. Infliximab and anakinra were also used, with 42% of respondents reporting anakinra use in patients with MAS, and 18% of respondents using infliximab for patients at high risk for CAAs (Figure 2A,B).

### 3.5. Treatment of IVIG-Refractory KD

**Figure 3:**
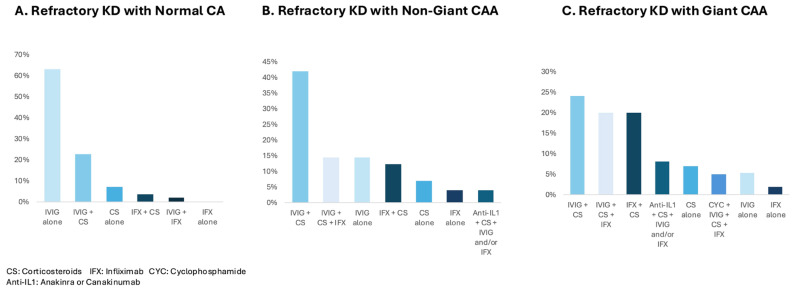
Variation in treatment of IVIG-refractory KD among responding pediatric rheumatologists. (**A**) Predominant use of IVIG with or without CSs in the absence of CAAs. (**B**,**C**) Increased practice variation in the presence of CAAs, with combinations of CSs, IVIG, and infliximab used most frequently.

The treatment of IVIG-refractory KD varied widely among respondents, particularly in the presence of CAAs (Figure 3). In a patient with KD without high-risk features, in the absence of CAAs, 63% of respondents would use a second dose of IVIG alone, 23% would use a second dose of IVIG together with CSs, 7% would use CSs alone, 4% would use the combination of CSs with infliximab, and 2% would use IVIG with infliximab. Third-line treatment choices for a KD patient with normal CAs and no high-risk features, who failed to respond to a second dose of IVIG or other immunomodulatory therapy, included CSs (77%), infliximab (57%; 4% as a second dose), anakinra (13%), a third dose of IVIG (11%), and cyclosporine (4%).

In the presence of non-giant CAAs, only 15% of respondents would use a second dose of IVIG alone; 40% would use a second dose of IVIG together with CSs. Infliximab was used by 35% of respondents, usually combined with IVIG plus CSs (15%) or just with CSs (12%). The use of CSs or infliximab alone was uncommon (7% and 4%, respectively), and the use of other medications, including anakinra or cyclosporine, was also uncommon.

For a patient with giant CAAs and IVIG-refractory KD, with no other high-risk features, the medications most frequently chosen by respondents were CSs, a second dose of IVIG, and infliximab (91%, 69%, and 58%, respectively), usually as a combination including two or more medications. The combination of IVIG plus CSs plus infliximab was reported by 33% of respondents (20% used this combination without other medications; 13% used at least one additional medication); 24% used a second IVIG dose plus CSs only, and 20% used CSs plus infliximab. Only 7% of respondents would use CSs alone, 5% would use a second dose of IVIG alone, and 2% would use infliximab alone. Anakinra, cyclophosphamide, and cyclosporine were added to treatment combinations by 9%, 9%, and 7% of respondents, respectively. The marked variation in treatment choices among respondents seen in patients with giant CAAs is illustrated in Figure 3. When the route and dose of CSs were considered, there were 32 different medication combinations.

In addition to the presence of CAA, respondents frequently identified several high-risk features of KD as factors leading to a different treatment choice for patients with IVIG-refractory KD. These included suspected MAS (83%), increasing CA dimensions (61%), COVID-19-associated KD (49%), age < 6 months (44%), and extreme elevation of inflammatory markers (30%). Other factors less frequently influencing the choice of treatment for IVIG-refractory KD included previous history of KD (21%), prolonged fever (>10 days) at presentation (16%), and age > 10 years at diagnosis (16%). The number of years respondents were in practice did not significantly influence treatment choices.

All respondents reported using CSs for IVIG-refractory KD at some point in time. Methylprednisolone was used both as pulse dosing (10–30 mg/kg/dose) and/or daily dosing (1–2 mg/kg/day). A total of 91% of respondents reported ever using infliximab for IVIG-refractory KD (dose 5 mg/kg (49%) or 10 mg/kg (56%)), and 72% reported ever using anakinra (dose 2–5 mg/kg/day (61%) or 6–10 mg/kg/day (33%)). Furthermore, 33% and 26% of respondents, respectively, reported ever using cyclosporine or cyclophosphamide for refractory KD. Only three respondents reported ever using canakinumab and one reported ever using etanercept.

### 3.6. Changes in KD Management Reported After the COVID-19 Pandemic

No change in the treatment of KD after the COVID-19 pandemic was reported by 65% of respondents, while 35% reported changing their management of KD patients. A total of 65% of these respondents reported increasing their use of CSs as the primary intensification of KD, along with IVIG, and 80% reported increasing their use of CSs for patients who failed to respond to a first dose of IVIG. The use of anakinra was reported more frequently by respondents who changed their management of KD after the COVID-19 pandemic compared with respondents who reported no change in practice. This included the use of anakinra as intensification therapy for KD complicated by MAS (60% vs. 32%), KD complicated by shock (20% vs. 8%), and KD associated with high-risk features for CAAs (20% vs. 8%). Respondents who had changed their practice also reported a higher frequency of anakinra use for the treatment of IVIG-refractory KD in patients with giant CAAs compared with those who had not changed their practice (20% vs. 3%).

## 4. Discussion

This survey examined practice variation in the treatment of KD by North American pediatric rheumatologists as a first step toward developing CTPs for patients with IVIG-refractory KD and enabling future comparative effectiveness research. Optimal management of patients who do not respond to the first dose of IVIG remains uncertain despite increased research in the past two decades. International guidelines agree on first-line treatment of standard-risk KD; however, they offer diverging recommendations for treating IVIG-refractory and high-risk patients [3,5,6,7]. In our survey, all respondents used IVIG and ASA as initial therapy; however, we found significant variation in the use of adjunctive therapies, both for primary intensification and for treatment of IVIG-refractory KD. Paucity of high-quality evidence may have led to this practice variability.

Most respondents would use primary intensification for patients with KD shock syndrome and macrophage activation syndrome, and over half would intensify initial treatment for patients with high-risk features for CAAs. Identifying high-risk patients is challenging in North America (NA), as criteria predictive of IVIG resistance in Japan may fail to identify patients who will develop CAAs in other populations [20,21]. In our survey, young age (<6 months) and the presence of CA abnormalities (LAD or RCA Z-score > 2.5) at the time of KD diagnosis were the risk factors most often leading to primary intensification. These are the most widely accepted predictors of CAAs in NA, and our findings likely reflect the 2021 ACR guidelines that conditionally recommend intensification with immunomodulatory therapy for these patients [5,22,23].

The choice of primary intensification therapy in patients at high risk for CAAs varied among respondents. CSs were used most often, followed by infliximab and anakinra. In a recent Cochrane review of eight studies assessing CS use in KD (seven as primary intensification), all studies favoring the use of CSs were carried out in Japanese patients at high risk for CAAs according to their scoring criteria [8]. Despite the lack of high-quality evidence supporting their use in other populations, CSs are included as options for primary intensification in both the 2021 ACR and the updated AHA 2024 KD guidelines [5,13]. Primary intensification using infliximab for patients at high risk for CAAs was reported by just under 20% of respondents. To date, no long-term benefit on CA outcome has been established for primary intensification with infliximab, although a shorter duration of fever has been reported [17]. Respondents used anakinra primarily in KD patients with MAS and less commonly in patients at high risk for CAAs without MAS. The contribution of IL-1 to the development of CA lesions in a KD mouse model and its role in systemic inflammation in KD suggest that anakinra may have a larger role in KD treatment in the future [24,25]. Cyclosporine was rarely used by respondents in our survey. Although the optimal choice of medication for primary intensification in patients with KD is still unknown, our study indicates that many pediatric rheumatologists believe that primary intensification is warranted for high-risk patients. This will be important to consider when developing a CTP for the treatment of IVIG-refractory KD, as it will directly impact subsequent treatment decisions.

Definitions of IVIG-refractory KD varied among respondents; over half defined IVIG-refractory KD as fever at 36 or more hours after completion of initial IVIG, consistent with the AHA guidelines [13], while another third of respondents made this diagnosis, at 24 h after IVIG completion. Other features commonly identified by respondents as indicating refractory KD and warranting additional therapy included CA abnormalities and a rising CRP after initial IVIG. IVIG-refractory KD patients require urgent treatment because they are at increased risk for CAAs. In our study, treatment of IVIG-refractory KD varied among respondents. For IVIG-refractory patients without CAAs or other high-risk features, most respondents used a second dose of IVIG alone, while a quarter reported using IVIG plus CS; other adjunctive therapies were used infrequently for this subgroup. This reflects published guidelines, where a second dose of IVIG with or without CS is the most common recommendation for IVIG-refractory KD despite limited evidence [3,5,6,26].

Our study identified several factors influencing the choice of treatment for IVIG-refractory KD, including the presence and size of CAAs, young age, extreme inflammation, and MAS. The presence of CAAs was a particularly important factor affecting responses. In patients with non-giant CAAs, a second dose of IVIG plus CSs was used most frequently, while a second dose of IVIG alone was used infrequently. One third of respondents reported using infliximab plus CSs, with or without a second dose of IVIG. The widest practice variation was reported in patients with giant CAAs. Respondents used numerous combinations of medications, most commonly including CSs, a second dose of IVIG, and infliximab. Anakinra, cyclophosphamide, and/or cyclosporine were used less commonly, and etanercept and canakinumab were rarely used. Usually, at least two treatments were combined.

Our study provides novel findings regarding the effect of the COVID-19 pandemic on the treatment of KD by pediatric rheumatologists in NA. Just over one third of respondents reported changing their management of KD following the pandemic; most reported increased use of CSs in patients with IVIG-refractory KD, and two thirds reported using CSs more frequently as primary intensification. In addition, respondents who reported changing their practice after the pandemic were more likely to use anakinra than those who reported no change, both for primary intensification and for IVIG-refractory KD. It is unclear whether the increased use of CSs and/or anakinra was based on concerns that patients with KD overlapped with MIS-C, or if this change was influenced by their experience treating MIS-C, a hyper-inflammatory syndrome with features similar to KD.

Importantly, we have identified several factors that should be considered when CTPs for IVIG-refractory patients are developed. These include the frequent use of primary intensification therapy, the influence of CAA size on treatment, and changes in KD management since the COVID-19 pandemic. Institution-specific protocols and access to echocardiography may also influence the use of CTPs. Engaging international cooperation will be important to enroll enough patients for comparative analysis. Fortunately, there are multiple KD groups committed to advancing care of these patients who have demonstrated a willingness to collaborate [27]. Limitations of our study include the small sample size and sampling bias. The survey only included pediatric rheumatologists who were members of CARRA in the USA and Canada; therefore, the results may not be generalizable to other countries or reflect treatment practices of other specialties, including cardiology, that care for KD patients. As with all surveys, the subjective nature of self-report is a limitation, and physician responses could not be confirmed by chart review. We could not determine whether therapeutic choices were made based on economic factors or physician experience rather than disease severity. We did use clinical vignettes, which have been shown to be a useful survey strategy [28].

## 5. Conclusions

We found significant variation in the treatment of KD by North American pediatric rheumatologists, including the use of primary intensification in high-risk KD patients as well as the treatment of IVIG-refractory KD, particularly in patients with CAAs. This practice variability is likely due to the lack of high-quality evidence to inform treatment choices. The current variation in treatment practices provides an opportunity to develop a limited number of consensus-derived treatment options (CTPs) whose effectiveness could be compared through research facilitated by an international registry, with the aim of identifying optimal therapy for IVIG-refractory KD.

## Data Availability

The original contributions presented in the study are included in the article/supplementary material, further inquiries can be directed to the corresponding authors.

## Supplementary Materials

The following supporting information can be downloaded at https://www.mdpi.com/article/10.3390/children12121695/s1: Survey questions (PDF version).

## Abbreviations

CARRA	Childhood Arthritis and Rheumatology Research Alliance
CER	Comparative Effectiveness Research
CA	Coronary Artery
CAA	Coronary Artery Aneurysm
CTP	Consensus Treatment Plan
IKDS	International Kawasaki Disease Symposium
IVIG	Intravenous Immunoglobulin
JIA	Juvenile Idiopathic Arthritis
KD	Kawasaki Disease
KDSS	Kawasaki Disease Shock Syndrome
LAD	Left Anterior Descending (Coronary Artery)
MAS	Macrophage Activation Syndrome
MIS-C	Multisystem Inflammatory Syndrome in Children
NA	North America
RCA	Right Coronary Artery
RCT	Randomized Control Trial

## References

[1] Day-Lewis M., Son M.B.F., Lo M.S. (2024). Kawasaki disease: Contemporary perspectives. Lancet Child Adolesc. Health.

[2] Tremoulet A.H., Best B.M., Song S., Wang S., Corinaldesi E., Eichenfield J.R., Martin D.D., Newburger J.W., Burns J.C. (2008). Resistance to intravenous immunoglobulin in children with Kawasaki disease. J. Pediatr..

[3] McCrindle B.W., Rowley A.H., Newburger J.W., Burns J.C., Bolger A.F., Gewitz M., Baker A.L., Jackson M.A., Takahashi M., Shah P.B. (2017). Diagnosis, Treatment, and Long-Term Management of Kawasaki Disease: A Scientific Statement for Health Professionals From the American Heart Association. Circulation.

[4] Chan H., Chi H., You H., Wang M., Zhang G., Yang H., Li Q. (2019). Indirect-comparison meta-analysis of treatment options for patients with refractory Kawasaki disease. BMC Pediatr..

[5] Gorelik M., Chung S.A., Ardalan K., Binstadt B.A., Friedman K., Hayward K., Imundo L.F., Lapidus S.K., Kim S., Son M.B. (2022). 2021 American College of Rheumatology/Vasculitis Foundation Guideline for the Management of Kawasaki Disease. Arthritis Care Res..

[6] Miura M., Ayusawa M., Fukazawa R., Hamada H., Ikeda S., Ito S., Kanai T., Kobayashi T., Suzuki H., Yamamura K. (2021). Guidelines for Medical Treatment of Acute Kawasaki Disease (2020 Revised Version). J. Pediatr. Cardiol. Card. Surg..

[7] de Graeff N., Groot N., Ozen S., Eleftheriou D., Avcin T., Bader-Meunier B., Dolezalova P., Feldman B.M., Kone-Paut I., Lahdenne P. (2019). European consensus-based recommendations for the diagnosis and treatment of Kawasaki disease—The SHARE initiative. Rheumatology.

[8] Green J., Wardle A.J., Tulloh R.M. (2022). Corticosteroids for the treatment of Kawasaki disease in children. Cochrane Database Syst. Rev..

[9] Kobayashi T., Saji T., Otani T., Takeuchi K., Nakamura T., Arakawa H., Kato T., Hara T., Hamaoka K., Ogawa S. (2012). Efficacy of immunoglobulin plus prednisolone for prevention of coronary artery abnormalities in severe Kawasaki disease (RAISE study): A randomised, open-label, blinded-endpoints trial. Lancet.

[10] Wang Z., Chen F., Wang Y., Li W., Xie X., Liu P., Zhang X., Zhang L., Huang P. (2020). Methylprednisolone Pulse Therapy or Additional IVIG for Patients with IVIG-Resistant Kawasaki Disease. J. Immunol. Res..

[11] Kobayashi T., Kobayashi T., Morikawa A., Ikeda K., Seki M., Shimoyama S., Ishii Y., Suzuki T., Nakajima K., Sakamoto N. (2013). Efficacy of intravenous immunoglobulin combined with prednisolone following resistance to initial intravenous immunoglobulin treatment of acute Kawasaki disease. J. Pediatr..

[12] Koné-Paut I., Tellier S., Belot A., Brochard K., Guitton C., Marie I., Meinzer U., Cherqaoui B., Galeotti C., Boukhedouni N. (2021). Phase II Open Label Study of Anakinra in Intravenous Immunoglobulin-Resistant Kawasaki Disease. Arthritis Rheumatol..

[13] Jone P.-N., Tremoulet A., Choueiter N., Dominguez S.R., Harahsheh A.S., Mitani Y., Zimmerman M., Lin M.-T., Friedman K.G. (2024). Update on Diagnosis and Management of Kawasaki Disease: A Scientific Statement From the American Heart Association. Circulation.

[14] Miyata K., Kaneko T., Morikawa Y., Sakakibara H., Matsushima T., Misawa M., Takahashi T., Nakazawa M., Tamame T., Tsuchihashi T. (2018). Efficacy and safety of intravenous immunoglobulin plus prednisolone therapy in patients with Kawasaki disease (Post RAISE): A multicentre, prospective cohort study. Lancet Child Adolesc. Health.

[15] Dionne A., Burns J.C., Dahdah N., Tremoulet A.H., Gauvreau K., de Ferranti S.D., Baker A.L., Son M.B., Gould P., Fournier A. (2019). Treatment Intensification in Patients With Kawasaki Disease and Coronary Aneurysm at Diagnosis. Pediatrics.

[16] Miyata K., Bainto E.V., Sun X., Jain S., Dummer K.B., Burns J.C., Tremoulet A.H. (2023). In fl iximab for intensification of primary therapy for patients with Kawasaki disease and coronary artery aneurysms at diagnosis. Arch. Dis. Child.

[17] Tremoulet A.H., Jain S., Jaggi P., Jimenez-Fernandez S., Pancheri J.M., Sun X., Kanegaye J.T., Kovalchin J.P., Printz B.F., Ramilo O. (2014). Infliximab for intensification of primary therapy for Kawasaki disease: A phase 3 randomised, double-blind, placebo-controlled trial. Lancet.

[18] Hamada H., Suzuki H., Onouchi Y., Ebata R., Terai M., Fuse S., Okajima Y., Kurotobi S., Hirai K., Soga T. (2019). Efficacy of primary treatment with immunoglobulin plus ciclosporin for prevention of coronary artery abnormalities in patients with Kawasaki disease predicted to be at increased risk of non-response to intravenous immunoglobulin (KAICA): A randomised controlled, open-label, blinded-endpoints, phase 3 trial. Lancet.

[19] Ringold S., Weiss P.F., Colbert R.A., DeWitt E.M., Lee T., Onel K., Prahalad S., Schneider R., Shenoi S., Vehe R.K. (2018). The Childhood Arthritis and Rheumatology Research Alliance Consensus Treatment Plans: Toward Comparative Effectiveness in the Pediatric Rheumatic Diseases. Arthritis Rheumatol..

[20] Davies S., Sutton N., Blackstock S., Gormley S., Hoggart C.J., Levin M., Herberg A.J. (2015). Predicting IVIG resistance in UK Kawasaki disease. Arch. Dis. Child.

[21] Jakob A., von Kries R., Horstmann J., Hufnagel M., Stiller B., Berner R., Schachinger E., Meyer K., Obermeier V. (2018). Failure to Predict High-risk Kawasaki Disease Patients in a Population-based Study Cohort in Germany. Pediatr. Infect. Dis. J..

[22] Salgado A.P., Ashouri N., Berry E.K., Sun X., Jain S., Burns J.C., Tremoulet A.H. (2017). High Risk of Coronary Artery Aneurysms in Infants Younger than 6 Months of Age with Kawasaki Disease. J. Pediatr..

[23] Son M.B.F., Gauvreau K., Tremoulet A.H., Lo M., Baker A.L., de Ferranti S., Dedeoglu F., Sundel R.P., Friedman K.G., Burns J.C. (2019). Risk Model Development and Validation for Prediction of Coronary Artery Aneurysms in Kawasaki Disease in a North American Population. J. Am. Heart Assoc..

[24] Gorelik M., Lee Y., Abe M., Andrews T., Davis L., Patterson J., Chen S., Crother T.R., Aune G.J., Rivas M.N. (2019). IL-1 receptor antagonist, anakinra, prevents myocardial dysfunction in a mouse model of Kawasaki disease vasculitis and myocarditis. Clin. Exp. Immunol..

[25] Kessel C., Koné-Paut I., Tellier S., Belot A., Masjosthusmann K., Wittkowski H., Fuehner S., Rossi-Semerano L., Dusser P., Marie I. (2022). An Immunological Axis Involving Interleukin 1beta and Leucine-Rich-alpha2-Glycoprotein Reflects Therapeutic Response of Children with Kawasaki Disease: Implications from the KAWAKINRA Trial. J. Clin. Immunol..

[26] Hatano S., Nakao H., Masuda H., Ono H., Kubota M., Ishiguro A. (2025). Effect of additional intravenous immunoglobulin for infliximab-refractory Kawasaki disease: A cohort study. Pediatr. Rheumatol. Online J..

[27] Rojas R.G., Breault F., Alzyoud R., Ayusawa M., Barri A.C., Belot A., Burns J.C., Choueiter N., Corinaldesi E., Fabi M. (2025). The landscape of inter-institutional and multinational collaborations in Kawasaki disease. Paediatr. Child Health.

[28] Veloski J., Tai S., Evans A.S., Nash D.B. (2005). Clinical vignette-based surveys: A tool for assessing physician practice variation. Am. J. Med. Qual..

