# Supplementary material for: Clinical Practice Variation Among Pediatric Rheumatologists Treating Kawasaki Disease: Results of a North American Survey
Source: Children (Basel). 2025 Dec 16;12(12):1695. doi: 10.3390/children12121695 (PMC12731380; doi:10.3390/children12121695)
Supplement: Supplementary file 1 [file children-12-01695-s001.zip › children-3931772-supplementary.pdf]

CARRA
Childhood Arthritis and Rheumatology Research Alliance

Refractory Kawasaki Disease Treatment Survey

Introduction

Dear CARRA member,

Thank you for completing this brief (approximately 10 min.) research survey about the treatment of IVIG-refractory Kawasaki Disease (KD). **If you are a practicing physician but do not provide care to children with KD, please still respond to the first 6 questions and then you will be exited from the survey.**

The best treatment to prevent coronary artery aneurysms (CAA) in KD patients who failed to respond to a first dose of IVIG (referred to as "IVIG refractory KD" in this survey) is currently unknown. The purpose of this survey is to help us develop consensus treatment plans (CTPs) for patients with IVIG-refractory KD. To determine if this is feasible, we need to understand your current treatment practices in patients with KD.

Please respond to this survey based on your practice, excluding patients who have Multisystem Inflammatory Syndrome in Children (MIS-C). The patients in this survey do not have MIS-C.

Your voluntary participation in this survey is greatly appreciated. Thank you in advance for your valuable time and assistance in this project.

The CARRA KD Working Group

(If you have any questions regarding this research survey, please contact either: Cagri Yildirim Toruner c.yildir@texaschildrens.org or Bianca Lang bianca.lang@dal.ca)

1. Please indicate if you would like to participate in this survey.

- ◯ Yes, I would like to continue with the survey
- ◯ No, I would like to opt out

Refractory Kawasaki Disease Treatment Survey

* 2. Are you currently a trainee?

- ◯ Yes
- ◯ No

Refractory Kawasaki Disease Treatment Survey

I. Background

I. Background

3. At your institution, which services care for the majority of children with KD. Please indicate primary or consultant service. (Please select all that apply)

	Primary	Consultant
Rheumatology	☐	☐
Hospital/General pediatrics	☐	☐
Infectious disease	☐	☐
Cardiology	☐	☐

Other (please specify)

4. Do you have specialty/subspecialty training, and in what discipline(s)? (Please select all that apply)

- ☐ No specialty training
- ☐ Pediatric rheumatology
- ☐ Adult rheumatology
- ☐ General pediatrics
- ☐ Infectious disease
- ☐ Cardiology
- ☐ Pediatric hospitalist
- ☐ Other (please specify)

5. How many years have you been in practice (after completion of training)?

- ≤5 years
- 6-10 years
- 11-20 years
- >20 years

* 6. Have you cared for children with IVIG-refractory KD as either the treating or consulting practitioner in the past 3 years?

- Yes
- No

CARRA
Childhood Arthritis and Rheumatology Research Alliance

Refractory Kawasaki Disease Treatment Survey

7. If yes, please estimate the number of IVIG-refractory KD patients you have provided care for in the last 3 years.

- ◯ ≤5
- ◯ 6-10
- ◯ 11-20
- ◯ >20

CARRA
Childhood Arthritis and Rheumatology Research Alliance

Refractory Kawasaki Disease Treatment Survey

8. At your institution, is IVIG-refractory KD treated according to an institution-specific protocol?

- ◯ Yes
- ◯ No
- ◯ Unknown

9. Please indicate echocardiography (ECHO) availability for patients with KD at your institution:

- ◯ Most of the time, ECHO result is available prior to the time of initial treatment of KD
- ◯ Most of the time, ECHO result is not available prior to the time of initial treatment of KD
- ◯ Echocardiography is not available at my institution; patients must be transferred or treated without echocardiographic information
- ◯ Unknown

10. In a patient with IVIG-refractory KD who responded to subsequent treatment, with normal coronary arteries (CAs) on initial ECHO, at what additional time points would you obtain follow up ECHOs? Please select all responses that apply.

- ☐ 1 week after initial diagnosis
- ☐ 2 weeks after initial diagnosis
- ☐ 4-8 weeks after initial diagnosis
- ☐ 12 months after initial diagnosis
- ☐ Unknown
- ☐ Other (please specify)

Refractory Kawasaki Disease Treatment Survey

II. Initial therapy of a child diagnosed with typical Kawasaki Disease

We will now ask for details concerning several stages at which therapeutic decisions are made when a child is admitted for KD.

Case: An 18-month girl presents with high fever for 5 days, bilateral non-purulent conjunctivitis, red cracked lips, a truncal rash and a 2 cm anterior cervical node. She is not hypotensive and does not appear acutely ill. Laboratory testing excludes infection and is consistent with a diagnosis of KD. COVID-19 testing is negative based on the tests available at your center. A pre-treatment ECHO was not done.

The patients in this survey do not have MIS-C.

11. In your current practice, which treatments would you administer to this child with typical KD as part of your **INITIAL** treatment of KD. Please select all responses that apply.

- ☐ Aspirin (High dose (>80mg/kg/d))
- ☐ Aspirin (Moderate dose (30-50 mg/kg/d))
- ☐ Aspirin (Low dose (3-5 mg/kg/d))
- ☐ IVIG (2g/kg)
- ☐ Glucocorticoids (oral)
- ☐ Glucocorticoids (IV daily)
- ☐ Glucocorticoids (IV pulse)
- ☐ Infliximab
- ☐ Etanercept
- ☐ Anakinra
- ☐ Canakinumab
- ☐ Cyclosporine
- ☐ Cyclophosphamide
- ☐ Other (please specify)

CARRA
Childhood Arthritis and Rheumatology Research Alliance

Refractory Kawasaki Disease Treatment Survey

III. “Intensified initial therapy" is sometimes given for children with KD who either present with severe illness or who are deemed to be at “high risk” of coronary artery aneurysms (CAA). The following two questions explore your use of "intensified initial therapy" for patients with KD (in order to define patient population for the CTP for KD).

12. Please indicate the circumstances listed below that would lead you to give "intensified initial thearpy" to a patient with KD. Please select all responses that apply.

- [ ] KD with suspected shock
- [ ] KD with suspected Macrophage Activation Syndrome (MAS)
- [ ] Age < or equal to 6 months old
- [ ] Age < or equal to 12 months old
- [ ] Age > 10 years old
- [ ] Prolonged fever (>10 days) at presentation
- [ ] Previous history of KD
- [ ] z-score 2 - 3 on echo prior to initial IVIG
- [ ] z-score >3 on echo prior to initial IVIG
- [ ] z-score > or equal to 10 on echo prior to initial IVIG
- [ ] I do not give "intensified initial therapy"
- [ ] Other (please specify)

13. If you do give **“intensified INITIAL therapy"** for children with KD with potentially severe disease or deemed at high risk of developing coronary artery aneurysms (CAA), which therapy do you typically select for patients in the categories below? Please select all treatments that apply for each category.

	KD with MAS	KD with shock	KD at high risk for CAA
Glucocorticoids (oral and/or IV)	☐	☐	☐
Infliximab	☐	☐	☐
Etanercept	☐	☐	☐
Anakinra	☐	☐	☐
Canakinumab	☐	☐	☐
Cyclosporine	☐	☐	☐
Cyclophosphamide	☐	☐	☐

Other (please specify)

CARRA
Childhood Arthritis and Rheumatology Research Alliance

Refractory Kawasaki Disease Treatment Survey

IV. Additional therapy for children with IVIG-refractory KD (i.e. patients who failed to respond to the first dose of IVIG without other immunomodulating treatment).

Determining which patients are considered refractory to initial IVIG

14. How many hours after the completion of the first dose of IVIG, would you consider presence of a fever (T $\geq$100.4^{o}F (38°C)) to signify IVIG-refractory KD? Choose one answer.

- ◯ 12 hours
- ◯ 24 hours
- ◯ 36 hours
- ◯ 48 hours
- ◯ Other (please specify)

15. Would you treat a patient with additional treatment following the first dose of IVIG on the basis of a fever of T $\geq$100.4^{o}F (38°C) alone (please assume no evidence of infection or suspected IVIG reaction)?

- ◯ Yes
- ◯ No

16. Which of the following clinical signs or laboratory abnormalities, if present after treatment with IVIG, would you use to determine that a patient is refractory to initial treatment and requires additional therapy? Please indicate if this feature alone would lead to additional treatment, or only in combination with fever. Please select all that apply.

	Alone	Only with fever	Never
**Persistent or worsening mucositis** at least 24 hours after treatment with IVIG	○	○	○
Recurrent/persistent **conjunctivitis** at least 24 hours after treatment with IVIG	○	○	○
Persistently elevated or **increasing WBC count** at least 24 hours after treatment with IVIG	○	○	○
Persistently elevated or **increasing CRP** at least 24 hours after treatment with IVIG	○	○	○
Presence or worsening of coronary artery abnormality	○	○	○
Other	○	○	○

Other (please specify)

____

Choice of treatment for patients who are refractory to initial IVIG

Case: Our 18-month girl with KD described previously, was treated with one dose of IVIG (2g/kg) and moderate dose aspirin with some improvement. However, fever (101.3^{o}F/38.5^{o}C) recurred 48 hours after completion of IVIG. Her conjunctivitis and red cracked lips also worsened. Her ECHO shows normal coronaries.

The patients in this survey do not have MIS-C.

17. What therapy would you use at this time in this patient with KD refractory to the first dose of IVIG? Please consider treatment if she has normal CAs, non-giant CA aneurysms (CAAs) or giant CAAs (GCAAs). The patients in this survey do not have MIS-C. Please select all responses that apply.

	Patient has Normal CA	Patient has non-giant CAA	Patient has giant CAA (GCAA)
No additional therapy	☐	☐	☐
Additional IVIG (2g/kg)	☐	☐	☐
Oral glucocorticoids (1-2mg/kg/day)	☐	☐	☐
IV daily glucocorticoids (1-2mg/kg/day)	☐	☐	☐
IV Pulse glucocorticoids (10-30mg/kg)	☐	☐	☐
Infliximab	☐	☐	☐
Etanercept	☐	☐	☐
Anakinra	☐	☐	☐
Canakinumab	☐	☐	☐
Cyclosporine	☐	☐	☐
Cyclophosphamide	☐	☐	☐

Other (please specify)

18. In your current practice, which of the following factors would have led to a different treatment choice for the treatment of IVIG-refractory KD? Please select all responses that apply.

- ☐ Age < or equal to 6 months
- ☐ Age > 10 years
- ☐ Prolonged fever at presentation (greater than or equal to 10 days)
- ☐ Extreme inflammation at presentation (very high CRP or ESR)
- ☐ Suspected MAS
- ☐ Previous history of KD
- ☐ CA dimensions increasing
- ☐ Suspected/ confirmed "COVID-19 related KD"
- ☐ Recent exposure to/infection with COVID-19
- ☐ None of the above
- ☐ Other (please specify)

V. Therapy for children with KD refractory to second dose of IVIG or other immunomodulatory treatment.

Case: Our 18-month girl with IVIG-refractory KD (2g/kg) was treated with your choice of additional immunomodulatory treatment. Her fever, red lips and conjunctivitis persist over the next 48 hours with no evidence/suspicion of infection including COVID-19. Her echo shows normal coronaries (the patients in this survey do not have MIS-C).

19. What therapy would you use at this time in this patient? Please select all responses that apply.

- [ ] No additional therapy
- [ ] Additional IVIG (2g/kg)
- [ ] Oral/IV glucocorticoids (1- 2mg/kg/day)
- [ ] IV Pulse glucocorticoids (10-30mg/kg/day)
- [ ] Infliximab
- [ ] Etanercept
- [ ] Anakinra
- [ ] Canakinumab
- [ ] Cyclosporine
- [ ] Cyclophosphamide
- [ ] Other (please specify)

VI. Medication use for patients with refractory KD

For the treatments listed below please indicate which you use and how you normally use them for refractory KD treatment. *Please select all responses that apply.*

20. Methylprednisolone IV

- [ ] Never used
- [ ] Pulse steroids (10-30mg/kg/dose)
- [ ] Moderate dose steroids (1- 2mg/kg/day)
- [ ] Other (please specify)

21. Prednisone/Prednisolone PO

- [ ] Never used
- [ ] 1- 2mg/kg/day (Maximum 60 mg/day)
- [ ] Other (please specify)

22. Etanercept

- [ ] Never used
- [ ] 0.8 mg/kg/dose SQ
- [ ] Other (please specify)

23. Infliximab

- [ ] Never used
- [ ] 5mg/kg/dose
- [ ] 10 mg/kg/dose
- [ ] Other (please specify)

24. Anakinra

- [ ] Never used
- [ ] 2-5mg/kg/day
- [ ] 6-10mg/kg/day
- [ ] Other (please specify)

25. Canakinumab

- ( ) Never used
- ( ) Used

26. Cyclosporine

- [ ] Never used
- [ ] 5-10 mg/kg/day
- [ ] Other (please specify)

27. Cyclophosphamide

- [ ] Never used
- [ ] 10 mg/kg IV
- [ ] Other (please specify)

28. Plasmapheresis

- ○ Never used
- ○ Used

29. Other:

Name:

Dose:

30. Has your treatment of a patient with typical KD changed as a result of the COVID-19 pandemic?

- ○ Yes
- ○ No

31. If your treatment of KD has changed since the pandemic, please indicate how. *Please select all that apply.*

- [ ] Increased use of steroids as initial treatment of KD along with first dose of IVIG
- [ ] Increased use of steroids for patients who failed to respond to a first dose of IVIG
- [ ] Other (please specify)

Refractory Kawasaki Disease Treatment Survey

VIII. Personal/Contact Information

* 32. May we contact you to clarify details or for further information?

- ◯ Yes
- ◯ No

CARRA
Childhood Arthritis and Rheumatology Research Alliance

Refractory Kawasaki Disease Treatment Survey

33. Contact Information

Name ______

Institution ______

Email Address ______

Phone Number ______

34. Please indicate your preferred method of contact.

- ○ Email address
- ○ Phone number

Refractory Kawasaki Disease Treatment Survey

Thank you very much for participating in this survey. Please click submit below to send your results.
